# 

**Published:** 1913-06-14

**Authors:** 


### The Lymphatic Diathesis.

For a good many years now the curious condition of "lymphatism," or the "lymphatic diathesis," has been described by physicians; but it was only when this pathological state began to be advanced in explanation of every other anæsthetic fatality in children that it attracted much attention. Inasmuch as in a fair proportion of these cases "lymphatism" undoubtedly had to bear the blame which might as reasonably have been apportioned elsewhere, intense scepticism sprang up concerning it; and, in the eyes of some doctors, lymphatism became an imaginary disease invented for the benefit of anæsthetists. This attitude is quite erroneous however; there is no question now that lymphatism does exist, and that its victims are really liable to sudden death on very slight provocation, especially on being anæsthetised. The difficulty has been, so far, to diagnose the condition during life. There are a large number of symptoms ascribed to the diathesis in the textbooks; but a diagnosis made before trouble has arisen has been a rare, though not by any means an unknown, occurrence. Now, however, Siess and Stoerk believe they have discovered a typical blood picture which enables the diagnosis to be made from a blood count. There is an increase, often very great increase, they say, in the number of blood plates; and, secondly, there is a decrease in the eosinophil corpuscles. The other elements of the blood remain normal. There is no lymphocytosis nor leucopenia. If this research is confirmed, the unravelling of the pathology of this complicated disorder will probably follow; and then we may get some practical information about the detection, prevention, and cure of lymphatism.

### Gifts and Bequests.

Mr. Franck Isaac Batchelor, of Hopwood, has bequeathed about £900 to local charities.

The Hampstead General and North-West London Hospital has received £50 from the Grocers' Company.

Mr. Charles Edward Boothby, formerly Ranger of Needwood Forest, has bequeathed £500 to the Royal Alexandra Hospital for Sick Children, Brighton.

Miss Mary Hall Pocock, of Reading, has bequeathed £1,000 to the Berkshire County Hospital, Reading, and £500 to the Royal Hospital for Incurables, Putney.

The Committee of Management of the Great Northern Central Hospital have received from the Trustees of Smith's (Kensington Estate) Charities the sum of £400.

Mrs. Caroline Sophia Phillimore, of Hurley Manor House, Berks, has left £105 to All Saints' Convalescent Hospital, Eastbourne; £150 to St. Mary's Convalescent Home, Broadstairs; and £200 to the Samaritan Hospital.

Mrs. Stanley Boulter, widow of Mr. D'Oyly Carte, the gross value of whose estate is £117,670, has bequeathed ten Five per Cent. Debentures of £100 each in the Savoy Hotel (Limited) to the Charing Cross Hospital.

The list of subscriptions received in connection with the centenary festival dinner of the London Orphan Asylum, Watford, has reached nearly £20,000, of which £2,500 has been subscribed by boys and girls who have been brought up at the institution.

Mr. George Southern, of Bamber Bridge, Lancashire, has left £1,000 each to the Preston and Lancashire Royal Victoria Infirmary and the Wigan Infirmary for the endowment of beds, and the residue of his estate, subject to various legacies and one life interest, to the Seamen's Orphanage, Liverpool; the Royal Cross School for Deaf and Dumb, Preston; the Homes for Blind, Fulwood; Dr. Barnardo's Homes; and such other charitable institutions as the trustees of his will shall determine.

### Appointments.

Dr. N. H. Turner, of Highbury Grove, N., has been appointed deputy medical officer of health for Islington and medical examiner to the Islington Borough Council in succession to Dr. J. A. Glover, who resigned the position on his recent removal to Hampstead.

### Buildings Contemplated.

Barrow-in-Furness.—The Guardians have been authorised to borrow £380 to purchase land for the extension of the infirmary.

Bucknall.—Extensions to the Stoke-on-Trent Isolation Hospital at Bucknall have been completed, and the new buildings formally opened. The expenditure involved amounted to £11,000.

Chartham.—Plans are in course of preparation for two new blocks at the Mental Hospital, Chartham, Kent.

Christchurch.—The Guardians have been authorised to borrow £3,753 for the building of a new laundry and the provision of machinery.

Colchester.—The new mental hospital for the county of Essex at Myland, Colchester, has been completed. It is anticipated that the cost, including furniture, will amount to nearly £500,000. The designs were prepared by Mr. F. Whitmore, the county architect, assisted by Mr. W. H. Town, of Colchester.

Cottingham.—A scheme has been adopted by the Hull Corporation Health Committee for erecting a sanatorium at Cottingham at an estimated cost of £17,367. The health committee recommend that for the male and female sanatoria and hospital blocks with dining-hall and kitchen, provided in the plans, the Frazzi construction be adopted. This form of construction consists of steel framing and terra cotta, with rough cast exterior and siraphite plaster interior. It is proposed to build the administrative portion in brick.

Dorset.—The County Council has appointed a committee to report upon the question of sanatoria accommodation.

Dublin.—The Corporation has adopted a scheme for dealing with tuberculosis in the city, which provides for a dispensary with one branch, a sanatorium, a hospital for advanced cases, and hospital accommodation for surgical cases. The Collier Dispensary has been purchased at a cost of £2,000.

Five Mile Town.—The Clogher Guardians have resolved to make application to the Local Government Board for sanction to a supplemental loan of £500 for the proposed medical officer's residence and dispensary.

Hinckley.—The Local Government Board have sanctioned a loan of £7,040 for a new hospital.

Liverpool.—The City Corporation of Liverpool is inviting designs for a sanatorium, which it is proposed to erect on a site at Fazakerley. Premiums of 150, 100, and 50 guineas respectively are offered for the first three selected designs.

Marlow.—The subscribers of the Marlow Cottage Hospital have decided to erect a new hospital, at a cost not exceeding £2,000, on a site offered by Mr. J. Langley.

Taylorton.—The Sterling County Council have passed a scheme submitted by the Central District Committee for a smallpox hospital at Taylorton.

Tendring.—The Rural District Council are considering the question of erecting an isolation hospital for the district.

Wellington, Salop.—The new Cottage Hospital is to be opened by Earl Powis on May 24. The hospital has been built in accordance with plans prepared by Mr. Leslie Moore, of Raymond Buildings, Gray's Inn, London.

Wolsingham.—The Durham County Council have decided to purchase Holywood Hall and estate, with the object of converting it into a sanatorium.

### Personalia.

The promotions and appointments to the Order of the Hospital of St. John of Jerusalem in England announced on Wednesday include as an Esquire W. Scatterty, Esq., M.D., D.P.H., from Honorary Associate.

Professor W. J. Simpson, C.M.G., left London recently for the East Coast of Africa, where he will conduct an inquiry in regard to plague and sanitary matters generally. During his absence Colonel W. G. King, C.I.E., will act for him.

Mr. F. E. Bullen has been presented with a fountain-pen by the London members of the National Union of Assistant Pharmacists, to commemorate his services as secretary. Mr. Bullen was pharmacist at the Brixton Dispensary for ten years.

We regret to announce the death of Mr. R. E. Jones, whose retirement we announced only last month. Mr. Jones has received only a few weeks' pension from the Poplar Board of Guardians, which body he served as dispenser for thirty-six years.

The King has been graciously pleased to appoint Colonel William Henry Bull, F.R.C.S. (Edin.), Assistant Director of Medical Services of the South Midland Territorial Division, and Colonel John Arnallt Jones, M.D., Assistant Director of Medical Services of the Welsh Territorial Division, to be Honorary Surgeons to his Majesty.

Dr. Peter Phillips Bedson, Mr. Robert Redhead, Mr. Dan Rolyat, and Mr. George Greener Elliott have been appointed honorary life governors of the Royal Victoria Infirmary, Newcastle. We recently noted that Mr. Dan Rolyat, who was seriously injured during a performance at the Tyne Theatre last year, paid the entire cost of his keep and treatment at the infirmary for three months, and generously subscribed £50 towards the funds in gratitude.

### Obituary.

Dr. Oscar William Clark, medical officer to Gloucester Prison, has died suddenly in the prison surgery. He had been medically attended a few days before. A graduate in natural science of Oxford, he became senior physician at the Gloucester Royal Infirmary, the staff of which he joined in 1885. He was president of the local branch of the British Medical Association.

Deputy Surgeon-General John Lloyd Thomas, R.N., has died. He retired in 1911 after an active career, during which he had been present at the fall of Port Arthur in 1894. He was also present during the Boxer Rebellion, and held the post of senior medical officer in charge of the Naval Brigade for the relief of Peking. He was promoted in 1896 to the rank of fleet surgeon, and served at Chatham, Bristol, and Liverpool.

Mr. James Henry Targett, F.R.C.S., has died in London, at the age of fifty-one. As senior examiner in midwifery under the Conjoint Board and pathological curator of the Museum of the Royal College of Surgeons for seven years, Mr. Targett enjoyed a high reputation. He studied at Guy's Hospital, and held several appointments, including, apart from house appointments, those of curator of the hospital museum, surgical registrar, and demonstrator of anatomy. He was an M.S. and M.B. of London University, obstetric surgeon to the Bolingbroke Hospital, and also ex-surgeon to the Samaritan Free Hospital for Women and to the Evelina Hospital for Children. He was also a frequent writer on medical topics.

### HOSPITAL SUNDAY FUND.

The following are among the principal amounts received at the Mansion House as the result of Hospital Sunday :—Mr. Heath Harrison, £300; Watts Watts & Co. (Limited), £210; Mr. Alexander Miller, £200; St. Peter's, Cranley Gardens, £195; Westminster Chapel, Buckingham Gate, £148; the City Temple, £137; St. Mary in the Boltons, S.W., £124; Brixton Independent Church, £102; Sir John Ramsden, Sir Walter Vaughan Morgan, Mr. W. D. Graham Menzies, Lieutenant-Colonel More Nisbet, and Mrs. Richard Foster, £100 each. The total available for distribution now amounts to about £29,000.

### FOREIGN GIFTS TO THE LISTER MEMORIAL.

The President of the Royal Society has received from the Portuguese Legation subscriptions amounting to £21 5s., forwarded by the Society of Medical Science of Lisbon to the Lister Memorial Fund; $867 has been collected by Dr. W. W. Keen, of Philadelphia. Further foreign donations include: University of Paris, 500f.; University of Lyons, 100f.; Société de Chirurgie of Lyons, 100f.; Faculty of Medicine of the University of Munich, 100m.; Faculty of Medicine of the University of Breslau, 110m.; and Stockholm Medico-Chirurgical Society, £5. A donation of £10 has been received from the University of Calcutta.

### Books Received.

Mills and Boon: "About Baby," by F. Tweddell and W. Barkley.

J. Wright and Sons: "Manual for Women's Voluntary Aid Detachments," by P. C. Gabbett, M.R.C.S.; "First Aid to the Injured and Sick," by Warwick and Tunstall; "Our Baby" (14th Edition, 100th Thousand), by Mrs. J. Langton Hewer.

